# Washout of pseudoexfoliation material combined with cataract surgery: a new surgical approach to lower intraocular pressure in pseudoexfoliation syndrome

**DOI:** 10.1007/s10792-014-9934-8

**Published:** 2014-04-05

**Authors:** V. Tao Tran

**Affiliations:** Centre for Ophthalmic Specialised Care, Clinic Montchoisi, Av. Beaumont 9, 1012 Lausanne, Switzerland

**Keywords:** Glaucoma, Pseudoexfoliation, Pseudoexfoliation syndrome (XFS), Pseudoexfoliation glaucoma (XFG), Cataract surgery, New washing technique, Special cannula

## Abstract

Glaucoma or ocular hypertension can be caused by the presence of pseudoexfoliation (PEX) material and/or pigmented cells in the trabecular meshwork (TM) and/or in the irido-corneal angle (ICA). Accumulation of this material can be highlighted by slit-lamp (SL), gonioscopy, and ultrasound biomicroscopy (UBM). Such material prevents aqueous humor from flowing out and thus induces intraocular pressure (IOP) elevation. A new technique using a special cannula for washing the TM and ICA, combined with cataract surgery, can lower IOP and reduce the number of hypotensive drugs needed. This study analyzed 11 patients (13 eyes) presenting a pseudoexfoliation glaucoma with cataract. They all had cataract surgery combined with the special washing technique. Visual acuity and IOP were noted before surgery, just after surgery and during follow-up. The number of hypotensive drugs needed was also recorded. Mean follow-up time was 34.4 months (range 21.8–59.2). The first case underwent surgery in 2007 and has a 5-year follow-up time. Local status was controlled by SL, gonioscopy, and UBM. Mean age was 79 years (range 71.6–86.0). Mean visual acuity was 0.37 pre-op (range 0.05–0.6) and 0.89 post-op (range 0.05–1.0). Mean IOP before and after surgery was 32.8 ± 8.7 mmHg (range 20–53) and 15.1 ± 3.5 mmHg (range 10–20), respectively. The amount of hypotensive drugs needed was 87 % lower after surgery. No PEX material recurrence was seen with SL, gonioscopy, and UBM during the mean follow-up of 3 years. No complication was recorded in this study. Cataract surgery combined with the new washout technique of the TM and ICA to remove PEX material or pigmented cells significantly lowers IOP and the amount of drugs needed. Long-term follow-up gives good results with no complication or recurrence. Eye status after surgery remains physiological and further glaucoma surgery can be performed if necessary. More research with a higher number of patients should be initiated to confirm this technique.

## Introduction

Dr. John G. Lindberg used the term “exfoliation” for the first time in 1917 [1]. Pseudoexfoliation syndrome (XFS) is a systemic disease characterized by the progressive formation and accumulation of fibrillar deposits in various tissues and organs [2, 3]. Ocular involvement in this syndrome is manifested by the chronic accumulation of an abnormal fibrillar matrix product or a complex of glycoproteins/proteoglycans [4] on the ciliary body, the zonules [5], the anterior surface of the lens [6], the iris, the edge of the pupil, the corneal endothelium, in the irido-corneal angle (ICA), on the trabecular meshwork (TM), and in Schlemm’s canal [7]. Although the physiopathology of this phenomenon is unclear, this anatomical distribution would support the hypothesis by which the origin of these microparticles is located at the level of the ciliary body. These pseudo-exfoliative particles are insoluble and follow the natural flow of aqueous humor to be finally deposited within the TM. These deposits slowly impede the physiological outflow of the aqueous humor and lead to intraocular pressure (IOP) elevation. In addition, this type of glaucoma is characterized by high-pressure fluctuations and responds incompletely and inconsistently to topical drug treatments. It exposes patients to an accelerated optic neuropathy progression and perimetric impairment.

In a defined population, the estimated overall age- and sex-adjusted annual incidence of XFS is 25.9 per 100,000 persons, while the same estimation of pseudoexfoliation glaucoma (XFG) is 9.9 per 100,000 persons. The incidence of both diseases is higher in females and increases with advancing age [8].

Upon clinical examination, gonioscopy reveals PEX deposits in the TM, usually at 6 o’clock on one or the other side of Schwalbe’s line. Examination by ultrasound biomicroscopy (UBM) can objectify these particles in the ICA, which are denser in the lower half [9].

Exfoliation material deposits contain lysosomal enzymes that lead to degeneration and dysfunction of the structures involved in the filtration process and these modifications are responsible for some pathological changes in the anterior segment such as zonular weakness, iridopathy, blood-aqueous barrier breakdown, ciliary body involvement, trabeculum impairment, and keratopathy [10].

Phacoemulsification is considered to be safe in most eyes with pseudoexfoliation even though significantly more complications occurred intraoperatively. The low frequency of inflammatory response indicates that the presence of pseudoexfoliation (PEX) does not significantly increase the risk of inflammation [11].

Glaucoma surgery is frequently indicated in the presence of PEX accompanied by ocular hypertension or glaucoma. The different techniques used, such as trabeculectomy, deep sclerectomy, viscocanalostomy, or trabecular aspiration are all performed [12], but all are associated with a high rate of complications, regardless of the technique chosen.

This study presents a new method combining cataract surgery with a simple and additional washout procedure of the TM and the ICA for patients with XFG and cataract. This eliminates the exfoliative particles and pigments located on the TM and in the ICA in order to restore the physiological pathway of aqueous humor either through the TM or via the ICA. Practically, the result of this IOP reduction is higher than the basic decrease of IOP post-op after phacoemulsification in eyes with XPS.

## Materials and methods

### Surgical method

The procedure starts with cataract surgery that should be slow and gentle, occasionally complemented by procedures such as staining of the capsule in case of opalescence of the cataract and iris hooks in case of a small pupil. Insertion of a capsular tension ring may reduce the peri- and post-operative risk. A larger capsulorhexis is useful to prevent anterior capsule opacification and contraction.

The following points are also important: (1) The use of bimanuality with two side-port incisions, one for infusion and other for aspiration; (2) The application of a water-jet hydrodissection with a flat-tipped needle without touching the anterior capsule to easily separate the core from the cortex without exerting too much pressure on the zonules of Zinn. The core, once liberated from the posterior capsule, can be turned on itself and fragmented into several parts, thereby reducing zonular traction. The aspiration of the cortex is also facilitated; (3) Finally, during the implantation of the intraocular lens, we renounce using viscogel, preferring BSS saline for inflating the capsular bag. This avoids washing viscogel at the end of surgery and helps in preventing a postoperative increase of IOP. A single tablet of Carbonic Anhydrase Inhibitor (acetazolamide 250 mg) was given to every patient 8 h post-op to prevent ocular hypertension (postoperative IOP spike) after washout.

Potentially, the complications of this new method include transient increase of IOP on the first post-op day, deformation of the pupil, thinning/atrophy or perforation of the iris and irido-dialysis due to the force of the water jet, the presence of hemorrhage from microvessels of the ICA and a cloud of pigment in the anterior or posterior chamber per-operatively, marking, peeling or tearing of the corneal endothelium due to direct contact with the cannula.

### Instrument: a new irrigation cannula

**Fig. 1:**
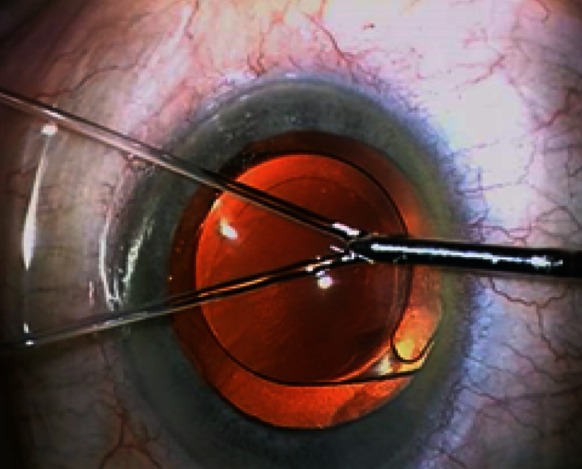
Cannula with two jets

**Fig. 2:**
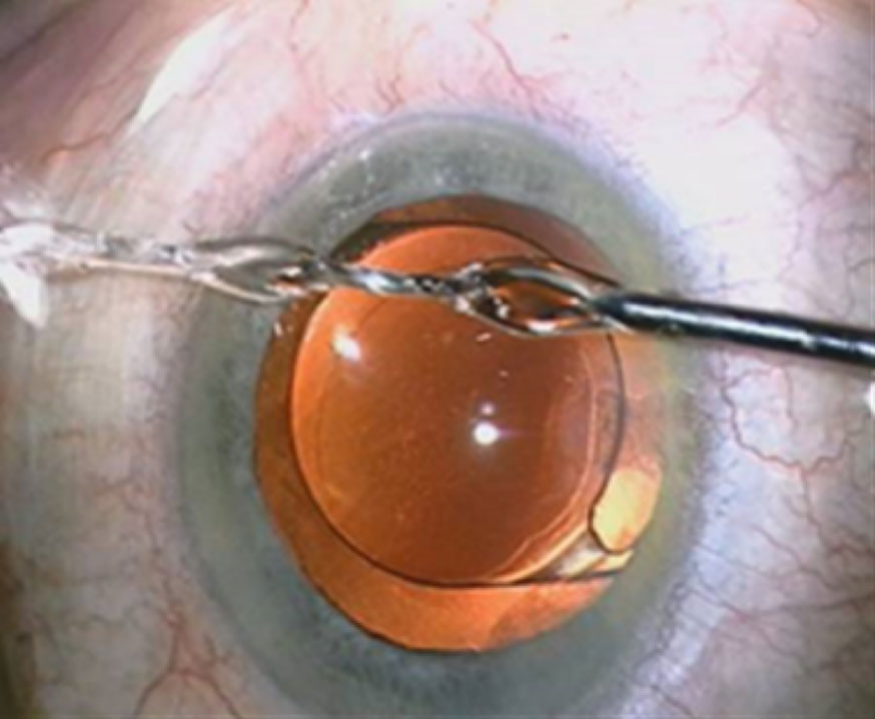
Cannula with only one jet after fusion

Following introduction of the implant into the capsular bag, we proceed to the second step: washing the pseudo-exfoliative particles. To achieve this step, we propose a new and original cannula capable of irrigating the ICA and TM. Unlike a conventional single jet irrigation cannula, this new instrument is characterized by two distinct water jets separated from each other by a 30° angle (Fig. 1). With the special design of this cannula and micro-hydrodynamic laws, these two independent jets can be merged into a stronger one (Fig. 2) when they encounter an obstacle in front of them. The two jets unify with its power depending upon the height of the bottle of BSS whose mean height is 90 cm higher than the patient’s eye. It would appear that this surprising and useful micro-hydrodynamic phenomenon of ocular microsurgery has never been described elsewhere.

Once merged into one water jet, we can now wash the TM and the ICA to remove the PEX material and pigment accumulation as much as possible.

Due to a greater accumulation of material in the lower quadrants, the washout time for the lower area is usually longer, lasting about two minutes in the 3 and 9 o’clock segments, and being about one minute for the upper area. The cannula should be placed directly but not close to the ICA to ensure optimal flow and avoid the contact with the corneal endothelium or the iris. Following a 360° irrigation of the ICA, the BSS cannula is used to clean other parts of the anterior chamber, in particular the edge of the pupil, the anterior surface of the iris, and optionally behind the iris. The jet force should be minimal in this area to avoid damaging the zonules, which are often weak in patients with PEX. Washing and rinsing of the ICA and the entire anterior chamber facilitate evacuation of most macro- and microscopic PEX deposits.

## Patients and methods

This study analyzed consecutive patients over a 5-year period (from 2007 to 2012) with XFG scheduled for cataract surgery seen at the Centre for Ophthalmic Specialised Care, Lausanne, Switzerland. All patients were treated according to the surgery technique described hereunder which is standard care in our centre since 2007, using the specially designed cannula, and were operated by the same surgeon (VTT).

All patients had a routine glaucoma work-up including best-corrected visual acuity, IOP measurement, visual field testing, and recording of glaucoma treatments before surgery, after surgery, and during follow-up. Best-corrected visual acuity was recorded using Snellen chart, IOP measurement was performed using Goldmann applanation tonometry. Slit-lamp and gonioscopy were performed in all patients. UBM was performed before and after surgery in the cases of presence of PEX material especially in the ICA and/or on the TM. The number of ocular hypotensive drugs needed was recorded before and after surgery. Demographic elements such as age and gender as well as ocular case history were recorded. Postoperative follow-up was carried out on day 1, day 5, and then at 1, 3, 6, 9 months and finally each semester upto 3 years on average. The continuation or discontinuation of topical or systemic ocular hypotensive drugs was noted, as well as the screening for possible complications or recurrence of PEX material in the usual places.

## Results

**Fig. 3:**
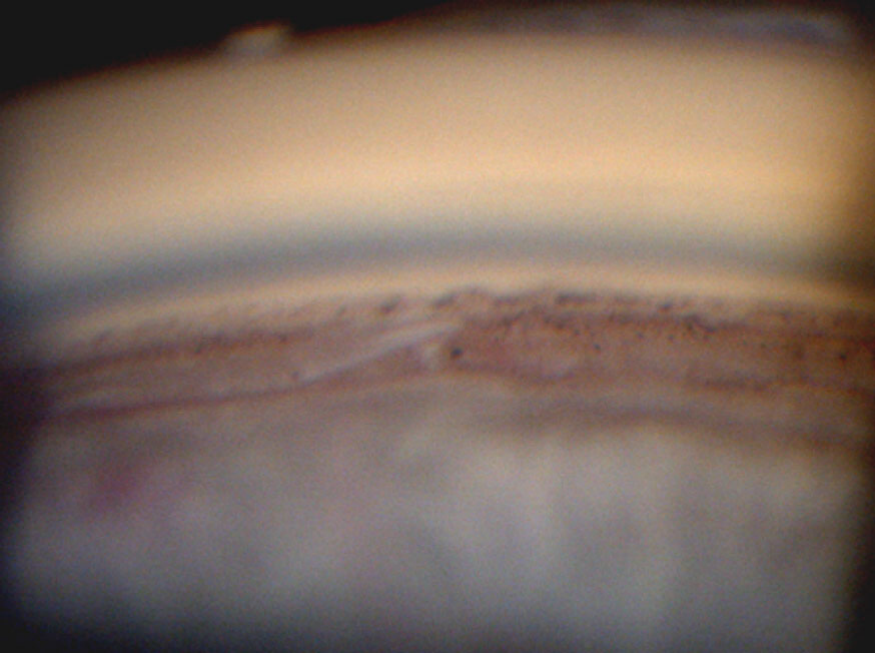
Presence of PEX on the trabecular meshwork and in the irido-corneal angle

**Fig. 4:**
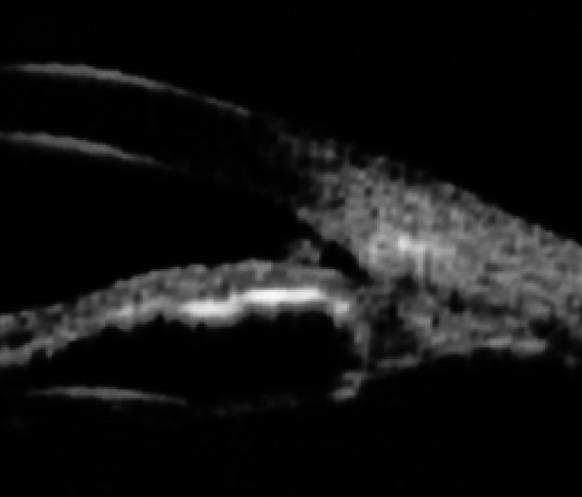
Presence of PEX in the angle objectified by UBM

**Fig. 5:**
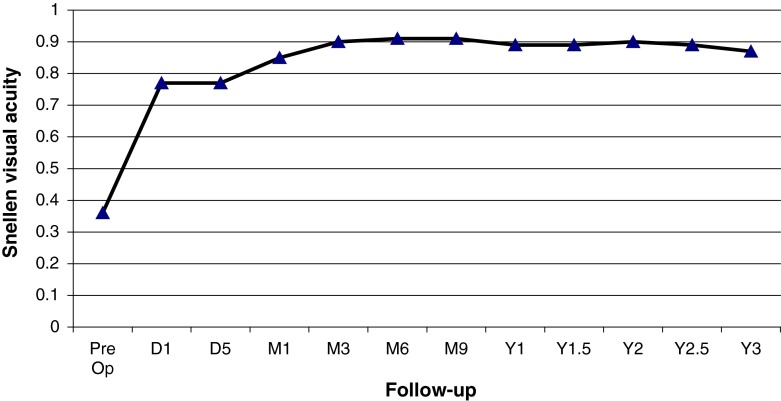
Follow-up of visual acuity (*D* day, *M* month, *Y* year)

**Fig. 6:**
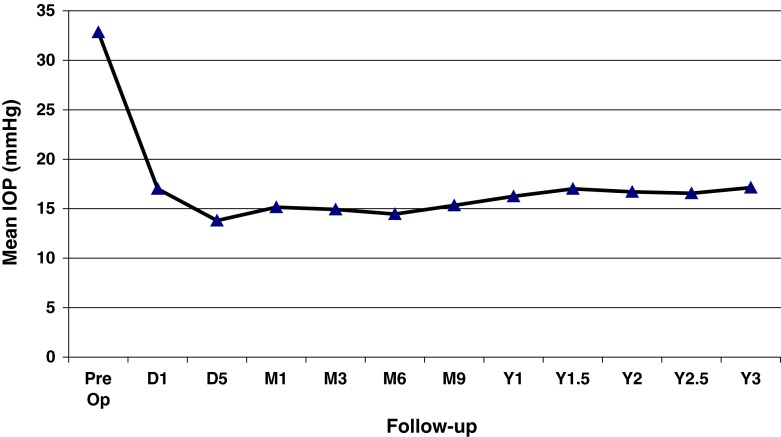
Follow-up of intraocular pressure (*D* day, *M* month, *Y* year)

From May 2007 to February 2012, eleven patients (13 eyes) were included with XFG and lens opacification requiring cataract surgery. PEX material in the ICA or on the TM was identified by UBM pre- and post-operatively (Figs. 3, 4). Mean age was 79 years (range 71.6–86.0). Mean visual acuity was 0.37 preoperatively (range 0.05–0.6) and 0.89 postoperatively (range 0.05–1.0). The follow-up of visual acuity remained generally stable during the three years postoperatively (Fig. 5). The mean IOP before and after surgery was 32.8 ± 8.7 mmHg (range 20–53) and 15.1 ± 3.5 mmHg (range 10–20 mmHg), respectively. IOP remained stable from the value at 1 month until the third year (Fig. 6). One eye had an IOP of 32 mmHg on the first day after surgery, probably explained by a very high-preoperative pressure (53 mmHg). Nevertheless, it returned to normal on the 5th post-surgical day and then rose after 1 year, requiring two topical hypotensive drugs. Three eyes required the addition of hypotensive eye drops after 1 year and 3 months in order to maintain acceptable values. The mean number of ocular hypotensive drugs (topical and systemic) was 2.38 before surgery and 0.31 after surgery, which means a decrease of 87 % after surgery. The follow-up duration was 34.4 months (range 21.8–59.2) with the first patient included in 2007. No complications and no new PEX deposits were observed during the mean 3 years of postoperative observation.

## Conclusions

This new technique combining cataract surgery with a simple additional washout procedure for patients with XFG respects the microanatomy of the eye because there is no additional incision, implant, or suture other than those required for the cataract surgery. This process tends to circumvent major complications of conventional glaucoma surgery. The added surgery time for this technique is short. It gives good long-term results with regard to both functional visual acuity and IOP. No complications have been observed in this study. The number of ocular hypotensive drugs was substantially reduced in post-op. Moreover, the scleral at 12 o’clock remained untouched and thus allows potential further glaucoma surgery. This work undoubtedly deserves a greater number of patients in order to confirm its therapeutic value.
